# Survey of alcohol-related admissions to critical care and the resource and financial implications

**DOI:** 10.1186/cc12432

**Published:** 2013-03-19

**Authors:** M Slattery, P Temblett, M Heatley, N Singatullina, B Jones

**Affiliations:** 1 ABMU University Trust, Swansea, UK

## Introduction

Alcohol-related hospital and ICU admissions are known to have a huge impact on healthcare resources in the UK. Excessive use of alcohol is independently associated with sepsis, septic shock and hospital mortality among ICU patients. This study assesses the relationship between alcohol abuse and intensive care resource utilisation in a mixed medical, surgical and neurosurgical ICU.

## Methods

A prospective survey of emergency alcohol-related admissions over a 1-year period was undertaken at a tertiary university adult general and neurosurgical ICU. All patients were screened for acute and chronic alcohol abuse on admission. Acute alcohol abuse was defined as being intoxicated with alcohol at the time of admission and chronic alcohol abuse was defined as chronic alcohol use exceeding recommended UK national guidelines on consumption. The amount of alcohol consumption was obtained, diagnosis on admission, ICU and hospital mortality, length of stay, and total cost were recorded. All patients were screened for alcohol-related comorbidities. Comparative retrospective data were obtained for the same time period for nonalcohol-related emergency ICU admissions. Data were analyzed using SPSS.

## Results

In total, 7.7% of patients were admitted with a history of acute/chronic alcohol excess. Sixty-seven per cent of alcohol-related admissions were due to acute alcohol excess. Neurosurgical patients admitted due to alcohol excess had higher ITU mortality than nonalcohol-related neurosurgical patients: 32.1% versus 14.39% (*P *= 0.02), respectively. Ninety-three per cent of alcohol-related neurosurgical admissions were caused by acute alcohol intoxication. The intensive care cost was significantly higher for alcohol-related (£12,396 per patient) compared with nonalcohol-related neurosurgical admissions (£7,284 per patient). Of the medical patients admitted, 60% of these admissions were due to acute alcohol excess. The cost of intensive care treatment was lower for alcohol-related medical admissions.

## Conclusion

This is one of the largest studies of alcohol-related admissions to critical care. Our survey confirms that alcohol-related admissions to the ICU are commonplace; however, our frequency is significantly less than previously reported. Our study reveals interspecialty variations in demographic data, APACHE II scores, mortality and cost of admission. Neurosurgical alcohol-related admissions bear higher mortality and result in greater resource utilisation relative to nonalcohol-related neurosurgical admissions. Alcohol continues to burden both our patients and critical care.

